# *Saccharomyces cerevisiae* mannan induces sheep beta-defensin-1 expression via Dectin-2-Syk-p38 pathways in ovine ruminal epithelial cells

**DOI:** 10.1186/s13567-019-0624-4

**Published:** 2019-02-04

**Authors:** Xin Jin, Man Zhang, Gui-fang Cao, Yin-feng Yang

**Affiliations:** 1 Veterinary Medicine College of Inner Mongolia Agricultural University, Hohhot, 010018 China; 2 Key Laboratory of Clinical Diagnosis and Treatment Technology in Animal Disease, Ministry of Agriculture, Hohhot, 010018 China

## Abstract

The rumen epithelium of sheep serves as an immune interface with the environment and secretes antimicrobial peptides with bactericidal function against various pathogens. Sheep beta-defensin-1 (SBD-1), an antimicrobial peptide, is secreted from ovine ruminal epithelial cells (OREC) in response to microbial infections. Mannan, the main component of the *Saccharomyces cerevisiae* cell wall can stimulate innate and regulatory immune responses that could improve the gastrointestinal environment. We aimed at investigating the effects of mannan on SBD-1 expression and the downstream signaling pathways stimulated in OREC. We cultured OREC; assessed the effects of mannan on SBD-1 expression by qPCR and ELISA; and then investigated the underlying signaling pathways using qPCR, ELISA, Western blotting, immunohistochemistry, and immunohistofluorescence. Interestingly, mannan markedly upregulated SBD-1 expression in a concentration- and time-dependent manner. Dectin-2 Mouse mAb, Syk specific inhibitor R406, and specific inhibitors of the p38, ERK1/2, JNK, and NF-κB pathways attenuated mannan-induced SBD-1 expression to varying degrees. These results demonstrate that SBD-1 is upregulated by mannan via the Dectin-2-Syk axis, and this is regulated to a large extent through the mitogen-activated protein kinase (MAPK) p38 and less so through the ERK1/2 and JNK or the NF-κB pathway. Our findings highlight the immunomodulatory effects of mannan on OREC in terms of mannan-induced SBD-1 expression.

**Electronic supplementary material:**

The online version of this article (10.1186/s13567-019-0624-4) contains supplementary material, which is available to authorized users.

## Introduction

Antimicrobial peptides are important components of the natural immune system, which play a key role in combating pathogen infections [1, 2]. Defensins, as an important member of antimicrobial peptides, are a family of small cysteine peptides with amphiphilic and cationic properties. These peptides are widely found in plants, insects, and vertebrates and are considered as components of the primitive and effective host defenses [3]. β-defensins represent one form of defensins that are principally produced by epithelial cells of various organs [4]. In addition to their direct antimicrobial activity, some β-defensins can also promote local innate and systemic adaptive immune responses [5, 6]. Only two kinds of β-defensins, SBD-1 and SBD-2, have been identified in ovine [7]. SBD-1 is an inducible peptide whose gene encodes 38 amino acid residues and is widely expressed in adult ovines [8]. Similar to human defensins, SBD-1 has a broad spectrum of antimicrobial activity against various bacteria, fungi, parasites, and some viruses in vitro; it exerts its bactericidal effect by electrostatically binding to negatively charged membranes and forming pores in bacterial cell membranes, thereby causing cell lysis [9]. Unlike SBD-1, which is widely expressed, SBD-2 is mainly expressed in the tongue, ileum, and colon of adult sheep [10].

Antibacterial peptides with short amino acid sequences can be synthesized by chemical methods [11, 12]. However, due to the high cost of chemical peptide synthesis, it may be a more effective alternative to induce defensin expression through dietary regulation. Previous studies have shown that β-defensins from different species can be induced by various yeasts and their cell wall components in vitro and in vivo [13–16]. The yeast cell wall is composed of multiple layers of carbohydrates, mainly including mannan and β-glucan [17]. Mannan is a bio-macromolecule derived from polysaccharides and can improve the host’s intestinal environment, regulate the intestinal microecological balance, induce the host’s intestinal immune response, and increase the animal’s humoral and cellular immunities [18, 19]. Furthermore, the mannan derived from *Candida albicans* reportedly stimulates IL-17 production in serum [20], increasing the transcription of β-defensin-2 mRNA in human keratinocytes [21].

Although mannan has been shown to induce defensin expression, its effects on SBD-1 expression in ovine ruminal epithelial cells (OREC) and the related mechanisms remain poorly understood. In 2010, Saijo et al. found that the mannan from *C. albicans* induced IL-1β and IL-23 secretion in a Dectin-2-dependent manner [22]. Dectin-2 is a newly discovered C-type lectin receptor, which is mainly expressed on the surface of various macrophages and dendritic cells. Dectin-2 has a mannose binding site, which can recognize the mannan structure of various microorganisms, such as *S. cerevisiae*, *C. albicans*, and *Mycobacterium tuberculosis*, and activate the downstream Syk and Card9-Bcl10-Malt1 signal adaptor proteins [23]. In turn, this induces the activation of the MAPK and NF-κB signaling pathways, which release a variety of cytokines, thereby effectively promoting the innate immune function [22–24]. The MAPK and NF-κB pathways play an important role in cell growth, differentiation, proliferation, and responses to various external stimuli [25–29]. Concurrently, MAPK and NF-κB family members control the expression of cytokines and anti-microbial effector genes. Therefore, they play an important role in physiological and pathological processes, such as inflammation and the immune response.

The present study is based on previous studies from our group [14]. We investigated the effect of *Saccharomyces cerevisiae* cell wall component, mannan, on SBD-1 expression and the mechanism underlying mannan-induced SBD-1 expression. Therefore, the results of this study provide a theoretical basis for the better development and utilization of mannan preparations. It is possible that dietary supplementation with mannan preparations may improve the innate immunity of ovines, which are vulnerable to diseases.

## Materials and methods

### Primary culture of ovine ruminal epithelial cells (OREC)

After obtaining approval by the Animal Ethics Committee of Inner Mongolia Agricultural University (License No. SYXK, Inner Mongolia, 2016-0015) ten adult Mongolian sheep (5 ewes and 5 rams, 7–12 months) were euthanized. After euthanasia, the rumen tissues (20 cm^2^) were harvested, rinsed with physiological saline, placed in ice-cold phosphate buffered saline (PBS) supplemented with 5% penicillin/streptomycin, and shipped to a biosafety cabinet for disposal.

All procedures were performed under aseptic conditions. The tissues were washed several times with PBS, and the mucosa was removed from the underlying epithelium and washed 6 times in PBS supplemented with 1 mg/mL penicillin, 500 μg/mL streptomycin, 100 μg/mL gentamicin, and 50 μg/mL amphotericin. The rumen mucosa tissues were subjected to 7 digestions by incubation with 0.25% trypsin at 37 °C for 45, 40, 30, 20, 15, 8, and 3 min, after which the digested products were observed under a microscope. A large number of small cells, which were mainly elliptical or circular with smooth edges and high refractive indexes, were observed after the third digestion. At this point, the cells were collected and trypsinization was stopped with DMEM/F12 (Gibco, Grand Island, USA) containing 20% fetal calf serum (FBS) (Gibco), and the above steps were repeated for subsequent cell collections. Next, the cell suspension was concentrated by centrifugation at 1000 rpm for 6 min, after which the cell pellet was washed twice with DMEM/F12 and then resuspended in DMEM/F12 supplemented with 20% FBS, 200 μg/mL penicillin, 100 μg/mL streptomycin, 5 μg/mL gentamicin, 2.5 μg/mL amphotericin, 2 μg/mL insulin-protein-selenium additive, and 2 μg/mL 2-mercaptoethanol. Finally, the cells were cultured in a 25-cm^2^ cell culture flask at 37 °C in a 5% CO_2_ incubator. The cells remained attached to the cell culture plates for more than 4 days, and the medium was replaced every 2 days.

### Induction tests

#### Screening for optimal induction concentration

After reaching 80–90% confluence, the OREC were passaged into 6-well flat-bottom culture plates containing DMEM/F12 medium, without FBS or antibiotics, and were incubated at 37 °C with 5% CO_2_ for a 24-h starvation treatment. Following this treatment, the OREC were then randomly divided into six groups: five mannan-stimulated groups and one control group. The stimulated groups were exposed to dose titration (10, 50, 100, 200, and 400 μg/mL) of mannan (Sigma, Munich, Germany), while the control group was cultured with DMEM/F12 medium instead of mannan at 37 °C, 5% CO_2_ for 8 h. Then, the total RNA of each sample was extracted, the cell culture supernatant was collected, and the expression levels of SBD-1 mRNA and protein were detected by qPCR and ELISA, respectively, to determine the optimal concentration of mannan-induced SBD-1 expression.

#### Screening for optimal induction time

The OREC were stimulated with the optimum concentration (50 μg/mL) of mannan for 2, 4, 8, 12, or 24 h in DMEM/F12 medium as the stimulated groups, while the control group received no mannan stimulation. Next, the total RNA of each sample was extracted, the cell culture supernatant was collected, and the expression levels of SBD-1 mRNA and protein were detected by qPCR and ELISA, respectively, to determine the optimal time of mannan-induced SBD-1 expression.

### OREC viability assay

Mannan toxicity was assessed by reducing 3-(4,5-dimethylthiazol-2-yl)-2,5 -diphenyltetrazolium bromide (MTT) to a methanol-soluble formazan product. OREC were seeded in 96-well plates at a density of 2 × 10^4^ cells per well. OREC treatments were performed for 8 h using mannan at concentrations of 10, 50, 100, 200, 400 μg/mL. Additionally, OREC were stimulated with the optimal concentration of mannan (50 μg/mL) for 2, 4, 8, 12, and 24 h. Medium alone was used as the control. Then, the OREC were washed thrice with PBS and cultured in DMEM/F12 containing MTT (5 mg/mL) for 4 h at 37 °C. The medium was then discarded and the plates were gently shaken for 10 min in 100 μL of DMSO to extract the formazan. The optical density of the formazan was determined using the Hybrid microplate reader (BioTek Inc., Winooski, VT, USA) at 540 nm. The effect of different concentrations of mannan on OREC viability was determined by calculating the ratio between treated and untreated OREC.

### Dectin-2 blocking experiments

For blocking experiments, cultured OREC were grown on 6-well flat-bottom culture plates and pretreated with Dectin-2 Mouse mAb (Abcam, Cambridge, UK) at varying concentrations (0.1, 1, 10 μg/mL) for 30 min, followed by activation with 50 μg/mL mannan for 4 h as the treatment groups; cells treated with mannan alone served as a positive control group, untreated cells served as a blank control group, and cells treated with only antibody, but not mannan, represented the negative control group. Next, the total RNA of each sample was extracted, the cell culture supernatant was collected, and the expression levels of SBD-1 mRNA and protein were detected by qPCR and ELISA, respectively.

### Inhibition tests

OREC were pretreated with the Syk-specific inhibitor R406 (1 μM and 5 μM) (InvivoGen, Toulouse, France) for 30 min, and the two different treatment groups were then stimulated with mannan. Cells stimulated with mannan, but not R406, represented the positive control. Cells treated with R406, but not mannan, represented the negative control, while untreated cells represented the blank control.

Then, the downstream pathways were inhibited using specific inhibitors: SB202190 (20 μM, Sigma) for p38, PD98059 (20 μM, Sigma) for ERK1/2, SP600125 (20 μM, Sigma) for JNK, and PDTC (10 μM, Sigma) for NF-κB. The cells were treated with the inhibitor for 60 min and the four different treatment groups were then stimulated with mannan (SB202190 + mannan, PD98059 + mannan, SP600125 + mannan, PDTC + mannan). Cells treated with only inhibitors (SB202190/PD98059/SP600125/PDTC) represented negative controls. Cells treated with mannan, but not inhibitors, represented the positive control, and untreated cells represented the blank control.

### Primers

The primers used to detect the expression of target genes (SBD-1, Dectin-2, Syk, p38, ERK1/2, JNK, NF-κB) in OREC treated with mannan are shown in Table 1. β-actin was used as a reference gene. The stable expression of this gene was validated using PCR and Western blotting. All primers were designed and synthesized by Sangon Biotech (Shanghai, China).

**Table 1:** **Primer sequences for qPCR**

Gene names	GenBank accession	Fragment size (bp)	Primer pair sequences (5ʹ–3ʹ)
β-Actin	U39357	208	F: GTCACCAACTGGGACGACA
			R: AGGCGTACAGGGACAGCA
SBD-1	U75250	133	F: GCTCTTCTTCGTGGTCCTGT
			R: ACAGGTGCCAATCTGTCTCA
Dectin-2	AM167931.1	146	F: GAGTGAGCAGAATTGCGTTG
			R: ATTGCCAGTTGCCATTCC
Syk	XM_004004064.3	139	F: GGAGGAGGCGGAAGACTACCTG
			R: CCTCTCGATGGTGTAGTGATGTGC
p38	NM_001142894.1	74	F: CGTTCAGTTCCTTATCTACCAG
			R: GCTCACAGTCTTCATTCACAG
ERK1/2	XM_012157699.1	90	F: GCGCTACACCAATCTCTCGT
			R: ATGGCGACTCGGACTTTGTT
JNK	XM_004002020.3	113	F: ATGACTGCAAAGATGGAAACGA
			R: ATGCTCTGCTTCAGAATCTTGG
NF-κB	XM_012159302.1	107	F: AGCACAAGAAGGCAGCACAA
			R: CCATCAGCAGCAGCAGACA

### RNA isolation, reverse transcription, and qPCR

Total RNA was extracted using the AxyPrep™ Multisource Total RNA Miniprep kit (Axygen Scientific Inc., Union City, CA, USA), according to the manufacturer’s instructions. The 260/280 ratio was measured using a Synergy H4 Hybrid microplate reader (BioTek Inc, Vermont, USA), and the RNA with an OD 260/280 ratio between 1.9 and 2.0 was reverse transcribed into cDNA using the PrimerScript™ RT reagent Kit with gDNA Eraser (TaKaRa, Tokyo, Japan). Following reverse transcription, the cDNA samples were analyzed by qPCR using the VIIA™7 Real-Time PCR System. Each 20 μL reaction consisted of 10 μL SYBR Premix Ex Taq (TaKaRa), 0.8 μL of each gene-specific primer (10 μM), 2.0 μL of cDNA template, and quantum-sufficient nuclease-free water. The thermal cycling consisted of 95 °C (30 s) and 45 cycles of 95 °C (5 s), 60 °C (34 s). Each condition was followed by a 46-step melt-curve analysis (95 °C for 5 s, 60 °C for 30 s, and 95 °C for 15 s). The relative mRNA abundance was calculated using the 2^−ΔΔCt^ method [30] and normalized to the mean expression of β-actin. Each experiment was repeated at least 3 times.

### ELISA detection of SBD-1 protein expression

Cell culture supernatants were respectively collected to determine the secretion of SBD-1 by OREC. The SBD-1 protein was quantified in the cell culture supernatants using the commercially available sheep defensin β1 (DEFβ1) ELISA kit (Wuhan Xinqidi Biological Technology, Wuhan, China) according to the supplier’s protocol.

### PCR detection of Dectin-2

For the PCR amplification of Dectin-2, PCR assays were performed in a final volume of 50 μL containing 25 μL of Premix Taq (TaKaRa), 22 μL of RNase-free dH_2_O, 1 μL of cDNA, and 2 μL of Dectin-2 primers (20 μM). The PCR amplification program started with a single denaturation step at 94 °C for 5 min, and 35 amplification cycles consisting of denaturation at 94 °C for 30 s, annealing at 60 °C for 30 s, and extension at 72 °C for 30 s, followed by a final extension at 72 °C for 7 min. The PCR products were analyzed by 1% agarose gel electrophoresis and Sanger paired-end sequencing.

### Immunohistochemical detection of Dectin-2 and Syk in rumen tissues of sheep

The rumen tissue sections were dewaxed in xylene followed by rehydration in a graded series of alcohol (100%, 95%, 85%, 70%, and 50%), and the antigen retrieval treatment was performed by microwave heating in 0.01 M citrate buffer (pH 6.0) and equilibration in PBS (pH 7.4). Both Dectin-2 and Syk were detected using the UltraSensitive™S-P Sensitive Kit (Rat/Rabbit) (Fuzhou Maxim Inc., Fujian, China) according to the manufacturer’s protocol. Immunoreactivity was detected using 3,3′-diaminobenzidine (DAB). Finally, sections were mildly counterstained with Mayer’s hematoxylin and observed under a light microscope. The primary antibody was Dectin-2 Mouse mAb (1:100) or Syk Rabbit mAb (1:200, Abcam), and an isotype-matched control antibody was used at equivalent concentrations. All images within each experiment were taken under the same conditions.

### Immunofluorescence detection of Syk and phosphorylated Syk in OREC

Cells were seeded on cover slips in 12-well plates at a density of 1 × 10^5^ cells/well. The next day, the cells were directly fixed with 4% paraformaldehyde for 30 min, permeabilized with 0.5% Triton for 20 min, and blocked with 5% bovine serum albumin for 1 h at 25 °C. The cells were stained with anti-Syk antibody (1:200) overnight at 4 °C, followed by staining with donkey anti-rabbit IgG H&L conjugated to Alexa Fluor^®^ 680 secondary antibody (Abcam) for 2 h at room temperature and counterstaining with DAPI. Cells were visualized with a LSM 800 confocal microscope (Zeiss, Oberkochen, Germany). All images within each experiment were taken under the same conditions.

### Western blotting detection of protein expression

Confluent cells were exposed to the indicated stimuli. After treatment, total protein was extracted with Western and IP cell lysates (Beyotime, Shanghai, China), according to the manufacturer’s instructions. The protein concentrations were measured using a BCA assay kit (Thermo, Waltham, MA, USA). Proteins were separated using SDS–polyacrylamide gel electrophoresis (PAGE) on 8-10% gels, and then transferred to polyvinylidene fluoride membranes (Millipore, Burlington, MA, USA). After the transfer, membranes were blocked for 2 h in 5% skim milk in Tris-buffered saline with 0.1% Tween-20 (TBST), and incubated overnight at 4 °C with primary antibodies Dectin-2 Mouse mAb (1:250), Syk Rabbit mAb (1:750), Phospho-Syk Rabbit mAb (1:750, CST, Danvers, MA, USA), IκB Mouse mAb (1:750, CST), Phospho-IκB Rabbit mAb (1:750, CST), p38 Rabbit mAb (1:750, CST), Phospho-p38 Rabbit mAb (1:750, CST), ERK1/2 Rabbit mAb (1:500, CST), Phospho ERK1/2 Rabbit mAb (1:500, CST), JNK Rabbit mAb (1:1000, CST), Phospho-JNK Rabbit mAb (1:1000, CST), p65 Rabbit mAb (1:750, CST), Phospho-p65 Rabbit mAb (1:500, CST), and β-actin Mouse mAb(1:1000, Beyotime). Following TBST washes, the membranes were incubated with the appropriate HRP-conjugated secondary antibodies (1:1000, Beyotime) in TBST. Finally, the membranes were visualized using a Signal Chemiluminescent Detection System (Chem Studio, Jena, Germany). The optical density of each band was analyzed using the Image-Pro Plus 6.0 software.

### Statistical analysis

All experiments were performed in triplicate and were repeated at least 3 times, and all data were plotted using the GraphPad Prism software and reported as the arithmetic mean ± SD. Comparison among multiple groups were made using a one-way ANOVA, and a comparison between two groups was made using an independent sample T test. Data analysis was carried out with SPSS version 20.0 (SPSS Institute Inc., Cary, NC, USA). Statistical significance was set at *P* < 0.05.

## Results

### Mannan induces SBD-1 mRNA and protein expression

Treatment with dose titration (10, 50, 100, 200, and 400 μg/mL) of mannan for 8 h caused a concentration-dependent increase in SBD-1 mRNA expression. SBD-1 expression was significantly upregulated when the cells were treated with 10 μg/mL mannan compared to the unstimulated cells (3.0-fold compared to the unstimulated group; *P* < 0.01), and the maximal induction was observed at 50 μg/mL mannan with a 5.8-fold increase in the SBD-1 mRNA expression (Additional file 1A). Moreover, the SBD-1 protein expression was consistent with its mRNA expression (Additional file 1B). Simultaneously, in order to determine the potential toxicity of mannan, OREC were treated with different concentrations of mannan for 8 h. The survival rate of OREC following mannan treatment was determined using the MTT assay. Notably, no mannan concentrations had any significant effects on cell viability compared to the unstimulated cells (*P* > 0.05; Additional file 1C).

Next, the optimal concentration of 50 μg/mL mannan was used for OREC stimulation in the subsequent experiments, in which OREC were treated for different time intervals (2, 4, 8, 12, and 24 h) to determine the kinetics of SBD-1 mRNA expression. The expression of SBD-1 mRNA was upregulated following a 2-h treatment compared to the unstimulated cells (5.2-fold compared to the unstimulated group at 0 h; *P* < 0.01), and the maximal induction was observed at 4 h, with a 13.4-fold increase in the SBD-1 mRNA expression, after which it declined (Additional file 1D). In addition, the results of SBD-1 protein was basically the same as its mRNA expression (Additional file 1E). Concurrently, the MTT assay showed that OREC viability was unchanged when exposed to 50 μg/mL mannan for 2, 4, 8, 12, and 24 h compared to the unstimulated cells (*P* > 0.05; Additional file 1F). In short, the expression of SBD-1 was the highest after 4 h following incubation with 50 μg/mL mannan, which suggests that these represent the optimal mannan concentration and incubation time for SBD-1 induction.

### Dectin-2 is expressed in OREC

**Figure 1:**
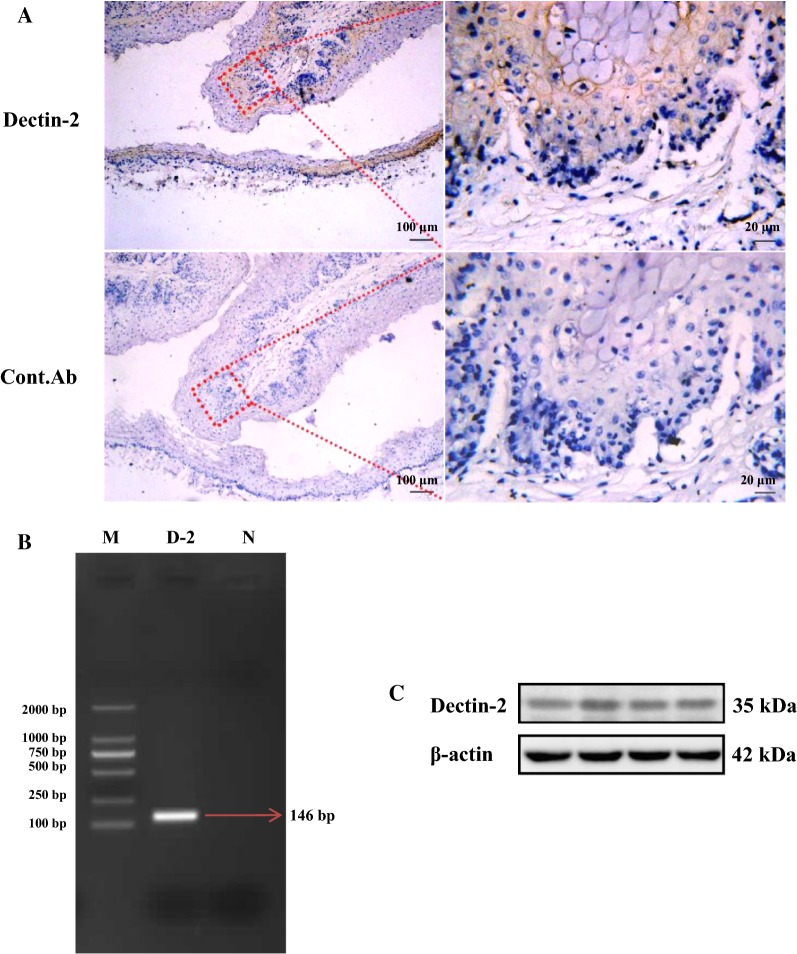
**Dectin-2 is expressed in ovine ruminal mucosa tissue and OREC. A** Dectin-2 expression in the spinous layer cells and basement layer cells of ovine ruminal mucosa tissue assessed by immunohistochemistry. Formalin-fixed paraffin-embedded sections of mucosa from the ovine rumen were stained with Dectin-2 Mouse mAb at 10 μg/mL. (Bottom panels) Staining with isotype-matched control antibodies served as a negative control. The picture on the right (40×) is an enlarged view of the red frame on the left side (10×). **B** Dectin-2 PCR results. Marker: D2000 DNA Marker; D-2: Dectin-2; N: Negative control. **C** Western blotting analysis of OREC cell lysates showing the Dectin-2 band (35 kDa). Blots were reacted with Dectin-2 Mouse mAb and with β-actin Mouse mAb as loading controls, and the four lanes in the figure represent four repetitions of the same lane.

To detect whether Dectin-2 is expressed in rumen tissue, paraffin-embedded sections generated from rumen tissues were stained with anti-Dectin-2 and isotype-matched control antibodies. Dectin-2 was mainly distributed in the spinous layer and basal layer of the rumen tissue mucosa (Figure 1A). Then, we used RT-PCR to further demonstrate that Dectin-2 is expressed in OREC; we observed a 146 bp PCR product, which was consistent with the expected fragment size (Figure 1B). The PCR product was purified and sent to BGI (Beijing) for paired-end sequencing, and the sequencing results were analyzed using BLAST (NCBI). The BLAST results show that the sequence had 100% homology with the ovine Dectin-2 mRNA (GenBank: AM167931.1, data not shown). Finally, a protein band of ~35 kDa was detected by Western blotting (Figure 1C). Taken together, these findings show that OREC express Dectin-2.

### Mannan induces SBD-1 expression via Dectin-2

**Figure 2:**
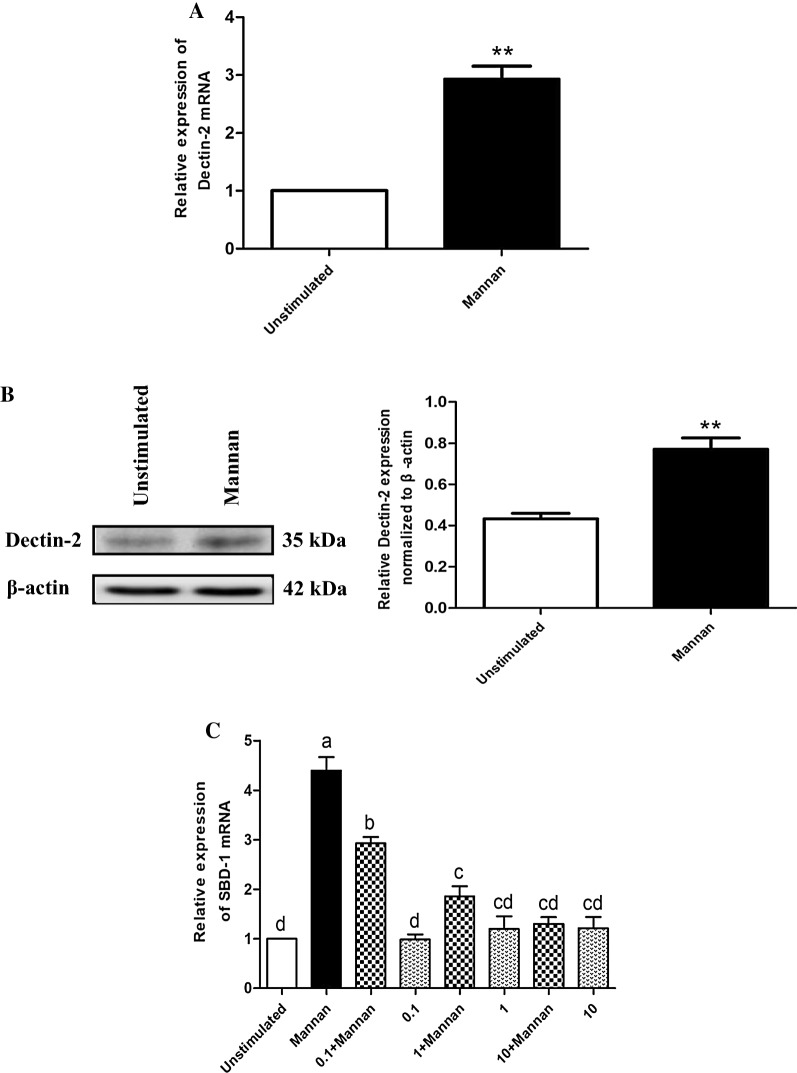
**Mannan induces SBD-1 expression via Dectin-2. A** Mannan stimulates Dectin-2 expression in OREC as measured by qPCR; the relative mRNA abundance was calculated using the 2^−ΔΔCt^ method relative to β-actin. Data are mean ± SD (*n* = 3). *P < 0.05, **P < 0.01 vs. the unstimulated group. **B** Mannan stimulates Dectin-2 expression in OREC as measured by Western blotting. Protein amounts are represented by the value shown in gray for the Dectin-2 protein/β-actin. Statistical analyses were performed using the ImageJ software. Data are mean ± SD (*n* = 3). *P < 0.05, **P < 0.01 vs. the unstimulated group. **C** The SBD-1 expression was determined by qPCR. OREC were incubated with Dectin-2 Mouse mAb (0.1, 1, 10 μg/mL) for 30 min prior to the addition of 50 μg/mL mannan for 4 h. Dectin-2 blockade by Dectin-2 Mouse mAb reduces mannan-induced SBD-1 expression in OREC. SBD-1 mRNA abundance was calculated using the 2^−ΔΔCt^ method relative to β-actin. Data are mean ± SD (*n* = 3). Different letters indicate significantly different means (*P* < 0.01).

To investigate the possible roles of Dectin-2 activation in mannan-induced SBD-1 expression, OREC were stimulated with mannan, and Dectin-2 expression was examined by qPCR and Western blotting. As shown in Figures 2A and B, treatment with mannan resulted in a significant increase in Dectin-2 mRNA and protein expression compared to the unstimulated cells (*P* < 0.01). The increased expression of Dectin-2 suggests that the Dectin-2 receptor can be activated by mannan.

We next wanted to determine whether the mannan-induced SBD-1 upregulation occurs via the common innate immune receptor, Dectin-2. SBD-1 mRNA and protein levels were significantly induced in response to mannan compared to the unstimulated cells (*P* < 0.01); but it could be attenuated by different concentrations (0.1, 1, 10 μg/mL) of Dectin-2 Mouse mAb (*P* < 0.05), and this effect became stronger as the Dectin-2 Mouse mAb concentration increased. Moreover, no significant differences were observed between the cells treated with only the antibody and the unstimulated cells (*P* > 0.05; Figure 2C and Additional file 2). In short, these results show that different concentrations of Dectin-2 Mouse mAb have no effect on cell viability and that Dectin-2 mediates the mannan-induced upregulation of SBD-1 expression.

### Syk is expressed in OREC

**Figure 3:**
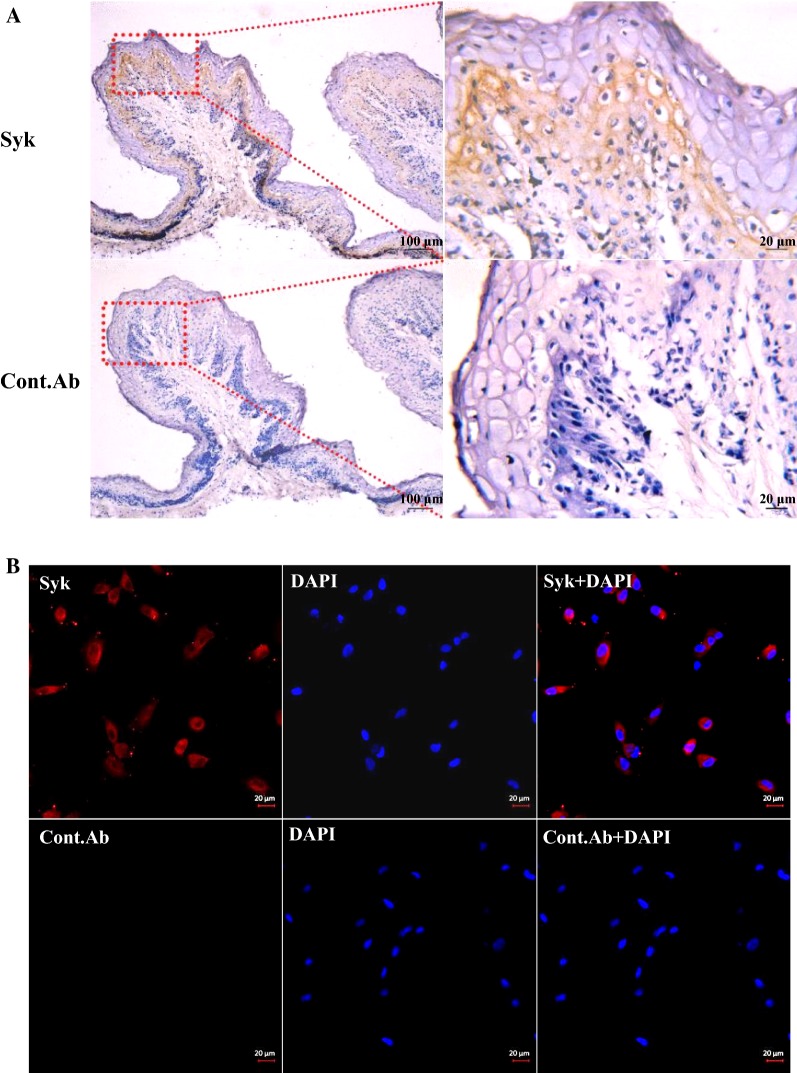
**Syk is expressed in ovine ruminal mucosa tissue and OREC. A** Syk expression in the ovine ruminal mucosa was assessed by immunohistochemistry. Formalin-fixed paraffin-embedded sections of mucosa from the ovine rumen were stained with Syk Rabbit mAb at 1.6 μg/mL. (Bottom panels) Staining of sections from the same blocks with isotype-matched control antibodies served as a negative control. The picture on the right (40×) is an enlarged view of the red frame on the left side (10×). **B** Syk expression in OREC detected by immunofluorescence. OREC seeded on cover slips were stained with Syk Rabbit mAb at 1.6 μg/mL, followed by staining with donkey anti-rabbit IgG H&L conjugated to Alexa Fluor^®^ 680 secondary antibody (red), and with DAPI (blue). (Bottom panels) Staining with isotype-matched control antibody.

After confirming that the Dectin-2 receptor is involved in the mannan-induced up-regulation of SBD-1 expression, we studied its downstream signaling adaptor, Syk. We investigated whether Syk, the cell signaling mediator of Dectin-2 activation, is expressed in OREC. Our immunohistochemistry analysis shows that the spinous layer and basal layer of the mucosal epithelium exhibited pronounced Syk expression (Figure 3A). Furthermore, the immunofluorescence results show that Syk was expressed in OREC (Figure 3B).

### The mannan-induced SBD-1 expression is Syk-dependent

**Figure 4:**
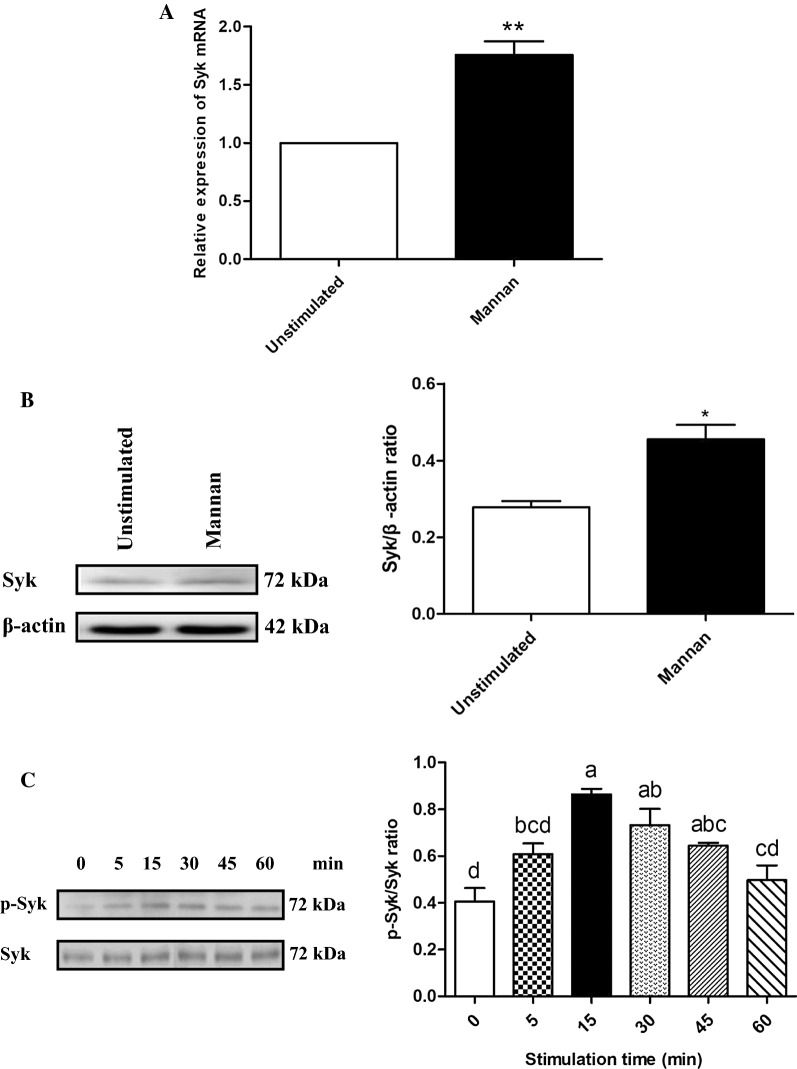
**Mannan-stimulated effect on Syk in OREC. A** Mannan stimulates Syk expression in OREC, as measured by qPCR; the relative mRNA abundance was calculated using the 2^−ΔΔCt^ method relative to β-actin. Data are mean ± SD (*n* = 3). *P < 0.05, **P < 0.01 vs. the unstimulated group. **B** Mannan stimulates Syk expression in OREC, as measured by Western blotting. Protein amounts are represented by the value shown in gray for Syk/β-actin. Statistical analyses were performed using the ImageJ software. Data are mean ± SD (*n* = 3). *P < 0.05, **P < 0.01 vs. the unstimulated group. **C** Expression of Syk and p-Syk was determined by Western blotting at 0, 5, 15, 30, 45, and 60 min after incubation with mannan. Data are mean ± SD (*n* = 3). Different letters indicate significantly different means (*P* < 0.01).

To investigate the possible role of Syk activation in mannan-induced SBD-1 expression, OREC were stimulated with mannan and the expression of Syk was examined by qPCR and Western blotting. Mannan stimulation resulted in a significant increase in Syk mRNA and protein expression compared to the unstimulated cells (*P* < 0.05, Figures 4A and B). This suggests that Syk can be activated by mannan. Simultaneously, OREC were stimulated with mannan for 0, 5, 15, 30, 45, and 60 min, and the phosphorylation of Syk was assessed by Western blotting. Syk was significantly phosphorylated at 15 min of mannan stimulation compared to the unstimulated cells (*P* < 0.01, Figure 4C). These results provide direct evidence for the role of mannan in the activation of Syk.

**Figure 5:**
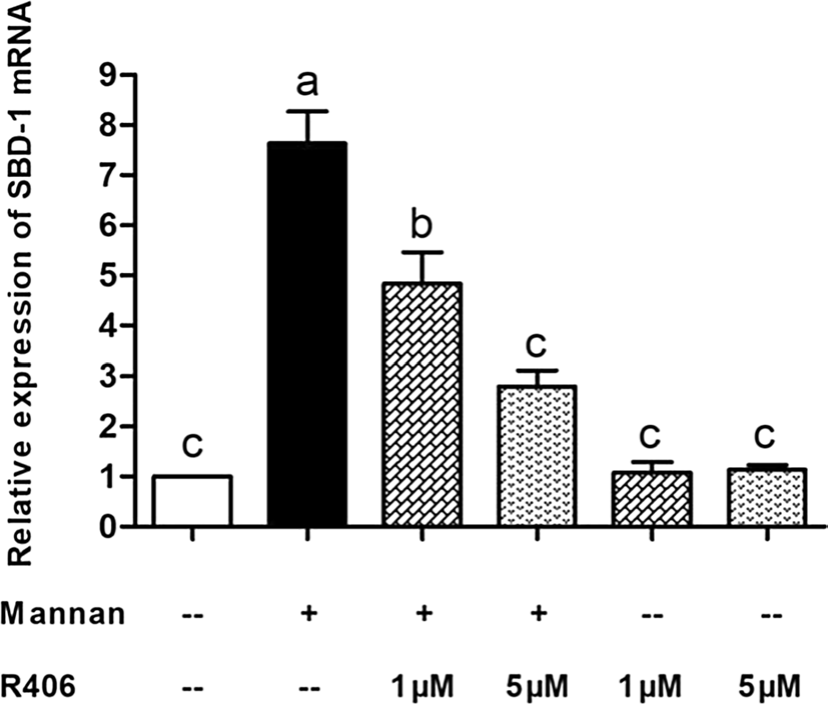
**Mannan-induced upregulation of SBD-1 is Syk-dependent.** SBD-1 expression, as determined by qPCR. OREC were incubated with R406 (1 μM and 5 μM) for 30 min prior to the addition of 50 μg/mL mannan for 4 h. Data are mean ± SD (*n* = 3). Different letters indicate significantly different means (*P* < 0.01).

We next determined whether the mannan-mediated SBD-1 upregulation involves Syk signaling. Mannan could significantly increase the SBD-1 mRNA and protein expression compared to the unstimulated cells (*P* < 0.01), and this expression could be decreased by the Syk-specific inhibitor R406 (*P* < 0.01). A 5-μM concentration of R406 significantly reduced SBD-1 expression by more than a 1-μM concentration (*P* < 0.01). Moreover, no significant difference was observed between the cells treated with only R406 and the unstimulated cells (*P* > 0.05, Figure 5 and Additional file 3). These results suggest that R406 (at the two concentrations: 1 μM and 5 μM) has no effect on cell viability and that Syk participates in the mannan-induced upregulation of SBD-1 expression.

### The activation of MAPK and NF-κB signaling pathways is mediated by Syk

**Figure 6:**
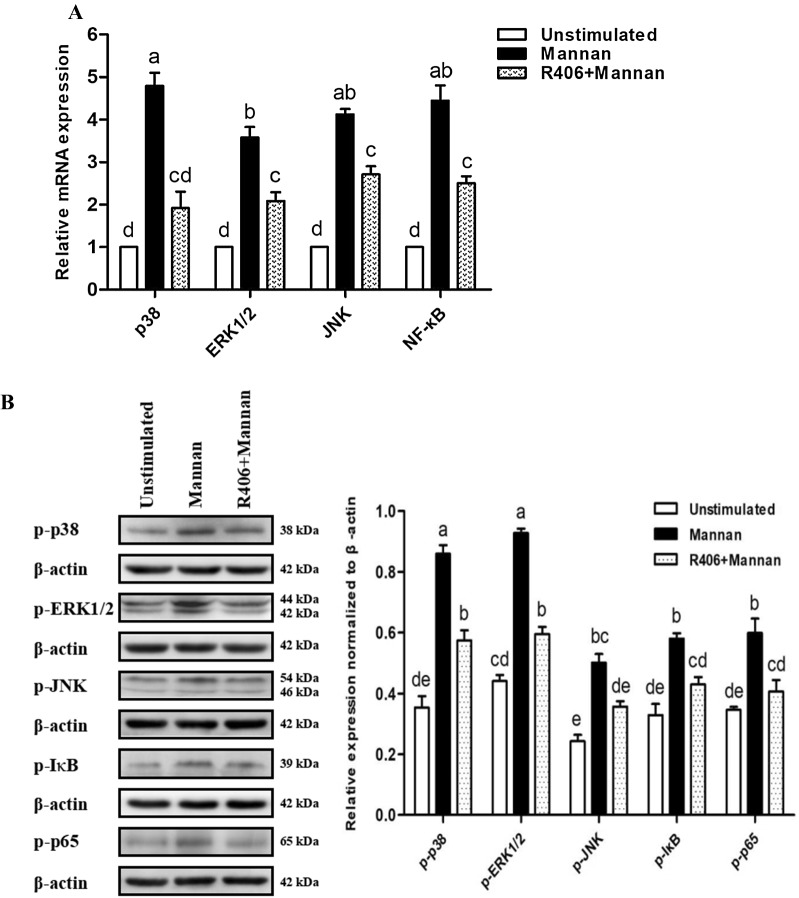
**The activation of MAPK and NF-κB signaling pathways is mediated by Syk. A** Detection of p38, JNK, ERK1/2, and NF-κB expression in OREC after mannan stimulation and R406 blockade by qPCR. The relative mRNA abundance was calculated using the 2^−ΔΔCt^ method relative to β-actin. **B** Protein amounts are represented by the value shown in gray for the target protein/β-actin. Statistical analyses were performed using the ImageJ software. Data are mean ± SD (*n* = 3). Different letters indicate significantly different means (*P* < 0.01).

Next, we investigated whether the activation of p38, JNK, ERK1/2, and NF-κB signaling in OREC was mediated by Syk. We used qPCR to detect expression changes in p38, JNK, ERK1/2, and NF-κB, and Western blotting to detect the phosphorylation of p38, JNK, ERK1/2, IκB and p65. The results show that mannan stimulation alone resulted in a significant increase in p38, JNK, ERK1/2, and NF-κB mRNA expression and p38, JNK, ERK1/2, IκB, and p65 phosphorylation levels compared to the unstimulated cells (*P* < 0.01). Moreover, pretreatment of OREC with the Syk inhibitor R406 (5 μM) for 30 min attenuated the increased mRNA expression of p38, JNK, ERK1/2, and NF-κB and the phosphorylation levels of p38, JNK, ERK1/2, IκB, and p65 induced by mannan (*P* < 0.01, Figures 6A and B). These results indicate that mannan induces SBD-1 expression via the Syk-mediated p38, ERK1/2, JNK, and NF-κB pathways in OREC.

### The role of MAPK and NF-κB in the mannan-induced SBD-1 expression

**Figure 7:**
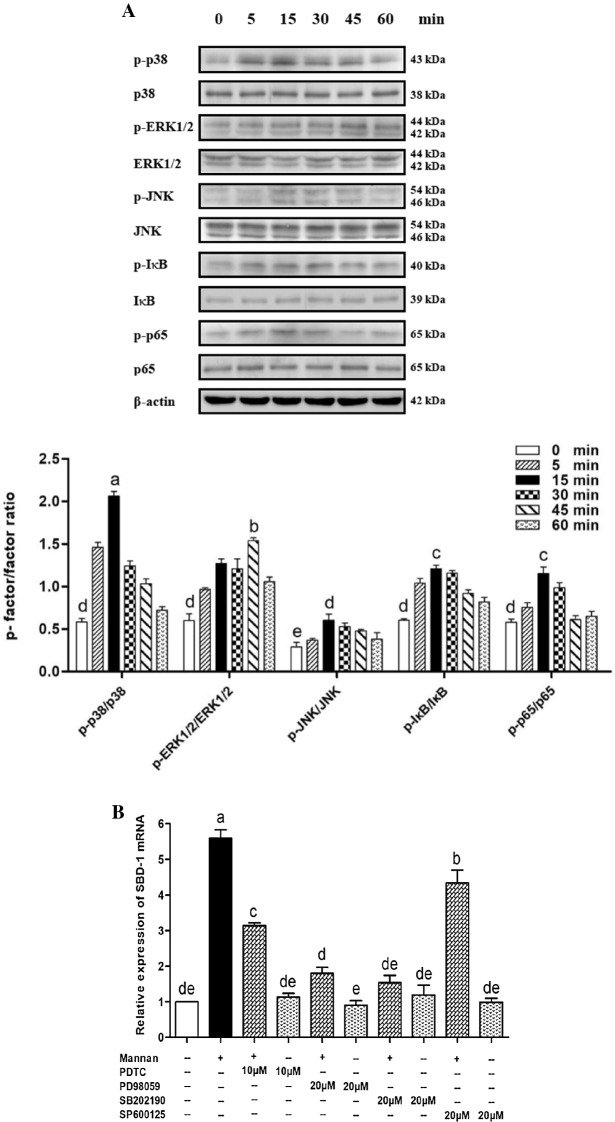
**Role of MAPK and NF-κB in the mannan induced SBD-1 expression in OREC. A** OREC were treated with mannan and harvested at 0, 5, 15, 30, 45, and 60 min. Whole-cell lysates were prepared and used for Western blotting analysis with p38 and p-p38, ERK1/2 and p-ERK1/2, JNK and p-JNK, IκB and p-IκB, p65 and p-p65 antibodies. Protein levels are represented by the value shown in gray for the p-factor/factor. Statistical analyses were performed using the ImageJ software. **B** Effect of inhibitors on the mannan-induced SBD-1 mRNA expression was determined by qPCR. OREC were cultured with mannan, with or without the SB202190 p38 inhibitor, PD98059 ERK1/2 inhibitor, SP600125 JNK inhibitor, and PDTC NF-κB inhibitor. The relative mRNA abundance was calculated using the 2^−ΔΔCt^ method relative to β-actin. Data are mean ± SD (*n* = 3). Different letters indicate significantly different means (*P* < 0.01).

To investigate the role of MAPK and NF-κB in the mannan-induced SBD-1 expression in OREC, we employed Western blotting. Interestingly, p38, ERK1/2, JNK, IκB, and p65 were activated after stimulation with mannan. The apparent phosphorylation of p38, ERK1/2, JNK, IκB, and p65 occurred at 5 min compared to the unstimulated cells (*P* < 0.05); they were markedly activated at 15, 45, 15, 15, and 15 min, respectively (*P* < 0.01), after which their levels began to decline. Overall, the maximum phosphorylation of p38, ERK1/2, IκB, and p65 was greater than that of JNK (*P* < 0.05). Moreover, the phosphorylation of p38 was significantly higher than that of ERK1/2, IκB, and p65 (*P* < 0.01) (Figure 7A). The above Western blotting results indicate that the p38, ERK1/2, JNK, and NF-κB pathways may be involved in the mannan-induced upregulation of SBD-1 expression. Next, qPCR and ELISA were employed to investigate the effects of p38, ERK1/2, JNK, and NF-κB inhibitors on SBD-1 mRNA and protein expression following mannan stimulation. Mannan significantly upregulated the expression of SBD-1 mRNA compared to the unstimulated cells (*P* < 0.01). Furthermore, the expression of SBD-1 mRNA induced by mannan was dramatically reduced in OREC pretreated with the p38 inhibitor SB202190, the ERK1/2 inhibitor PD98059, the JNK inhibitor SP600125, and the NF-κB inhibitor PDTC (*P* < 0.01), but the inhibition ability of the 4 inhibitors was different; the inhibitory capacity of SB202190 and PD98059 was significantly higher than that of PDTC and SP600125 (*P* < 0.01) (Figure 7B). The ELISA results (Additional file 4) demonstrate that SBD-1 protein levels were consistent with the observed mRNA levels. Pretreatment with SB202190 significantly decreased the mannan-induced SBD-1 expression, followed by PD98059 and PDTC. SP600125 also slightly regulated SBD-1 expression, and the inhibitory capacity of SB202190 was significantly higher than that of PD98059 (*P* < 0.05). These results suggest that the p38, ERK1/2, JNK, and NF-κB pathways mediate the mannan-induced upregulation of SBD-1 expression and that p38 in the MAPK pathway may constitute a key signaling axis.

## Discussion

Defensins are endogenous peptides that have important antimicrobial effects in vivo. With the development of biological sciences, the use of defensins as disease-preventing treatments has become a new therapeutic intervention. Our previous studies have shown that *Saccharomyces cerevisiae* can induce the expression of SBD-1 and that yeast affects the immune system mainly through its cell wall components [31]. For example, β-glucan, a yeast cell wall component, has been shown to increase β-defensin expression in scallops, fish, chickens, and humans [15, 32–34], and our team also found that β-glucan from *Saccharomyces cerevisiae* can up-regulate the expression of SBD-1 in OREC [35]. However, the effect of another *Saccharomyces cerevisiae* cell wall component, mannan, on the expression of SBD-1 has not been reported. Therefore, we stimulated OREC with mannan to detect its effect on the expression of SBD-1 and the underlying mechanisms.

This study found that SBD-1 was induced by mannan in a concentration- and time-dependent manner, and was decreased when the mannan concentration was too low or too high. In 2012, Wen et al. [36] found that SBD-1 expression in ovine oviduct epithelial cells (OOECs) is modulated by 17β-estradiol in a concentration and time-dependent manner and that the levels of SBD-1 were the highest when OOEC were stimulated for 3.5 h with a concentration of 10^−8^M. Similarly, in 2016, Li et al. [37] found that LPS regulates SBD-1 mRNA expression levels in OOEC in a similar fashion and that the levels of SBD-1 were the highest when OOEC were stimulated for 12 h with a concentration of 100 ng/mL. Recent studies have reported that β-glucan induces the upregulation of SBD-1 in OREC in a concentration and time-dependent manner and that the levels of SBD-1 were the highest when OREC were stimulated for 2 h with a concentration of 10 μg/mL [35]. Although all of these studies are concentration and-time dependent, the concentrations of irritants and stimulation times are different. These differences may be due to the differences in the cells stimulated and the different stimuli applied. Moreover, an appropriate mannan concentration and stimulation interval can induce a high SBD-1 expression in OREC. If this also occurs in vivo, SBD-1 may possess extracellular functions. Innate immunity may increase following an increase in SBD-1 expression in the body, while the mannan-induced upregulation in SBD-1 expression may also represent a type of regulatory process in the body. Thus, when the expression of SBD-1 reaches a peak following mannan induction, the body will gradually adjust SBD-1 expression to the basal level by lengthening the stimulation time, thereby maintaining the dynamic balance of SBD-1.

Since mannan could induce SBD-1 expression in OREC, we attempted to unravel the mechanism underlying this activation. Dectin-2 was recently shown to be the functional receptor for mannan, whose recognition leads to activation of the host innate immune system [19]. Previous studies have shown that Dectin-2 mRNA expression was found in the bovine small intestine and abomasum [38]. However, the expression of Dectin-2 in the rumen of sheep has not been reported. Here, we show for the first time the expression of Dectin-2 in the rumen tissue of sheep, and this expression is localized to the mucosal epithelial layer of rumen tissue. Dectin-2 expression in the rumen implies that in vivo fungal recognition by OREC is possible. Studies have shown that adding mannan from *Candida albicans* to peripheral blood mononuclear cells of mice slightly increase the mRNA level of Dectin-2 [39]. In this study, it was found that Dectin-2 was also expressed in cultured OREC and that mannan could up-regulate the expression of Dectin-2 in OREC. Hence, it is reasonable to assume that Dectin-2 might be functional in epithelial cells other than immune cells and could participate in mannan-induced responses in rumen mucosal surfaces. In addition, the addition of mannan to macrophages induces differentiation of Th17, and this Th17 differentiation was abrogated when macrophages lacked Dectin-2, indicating the critical role of Dectin-2 in macrophages for mannan-induced donor Th17-differentiation [40]. We found a marked increase in the expression of SBD-1 in response to mannan. This was significantly reduced by the Dectin-2 Mouse mAb, indicating that Dectin-2 is involved in the mannan-induced expression of SBD-1 in OREC.

In immune cells, Dectin-2 is coupled to the Syk kinase, which becomes phosphorylated upon Dectin-2 activation, and further activates the innate immune and host defense responses [41, 42]. In our study, Syk expression in the rumen mucosa and OREC was detected by immunohistochemistry and immunofluorescence, and the localization of Syk in OREC was consistent with that in human bronchial epithelial cells [43]. This represents the first account of Syk expression in OREC. In 2017, Lamprinaki et al. [44] showed that mouse bone marrow-derived dendritic cells produced the inflammatory cytokines, TNFα and IL-1β, in response to yeast mannan in a Dectin-2 and Syk-dependent manner. Our study also found that Dectin-2 and Syk are involved in the mannan-induced SBD-1 expression. Therefore, Dectin-2 is an important host receptor that can recognize mannan in the yeast cell wall and trigger an immune response via Syk. This interaction leads to the activation of host signaling cascades and the production of SBD-1. SBD-1, as an immune signaling molecule, can regulate the rumen mucosal immune response, thereby enhancing the body’s immune system, which can further activate the adaptive immune system.

Recent studies have confirmed that the MAPK and NF-κB pathways mediate the expression of antimicrobial peptides in mammalian epithelial cells through various stimuli [45, 46]. Therefore, the potential signal transduction mechanisms governing the mannan-induced upregulation of SBD-1 expression in OREC were investigated. We observed that the phosphorylation of p38, ERK1/2, JNK, IκB, and p65 in OREC following mannan induction were time-dependent and initially increased and then decreased. These results indicate that the mannan-induced upregulation of SBD-1 expression is a process that requires the activation of MAPK and NF-κB pathways. Moreover, by pretreating with specific p38, ERK1/2, JNK, and NF-κB inhibitors, we found that these pathways mediate the SBD-1 expression induced by mannan, with p38 representing the major signaling pathway. These observations are consistent with the lipopolysaccharide-induced SBD-1 expression in ovine oviduct epithelial cells (OOEC), which is also mediated by the p38 pathway [37]. In contrast, 17β-estradiol-mediated increase in SBD-1 expression in OOEC is mediated exclusively via NF-κB [36]. Therefore, SBD-1 expression in different tissues is likely mediated by different signaling pathways. This may be due to differences in specific stimulants and tissues, but the explicit reasons require an in-depth, follow-up study.

**Figure 8:**
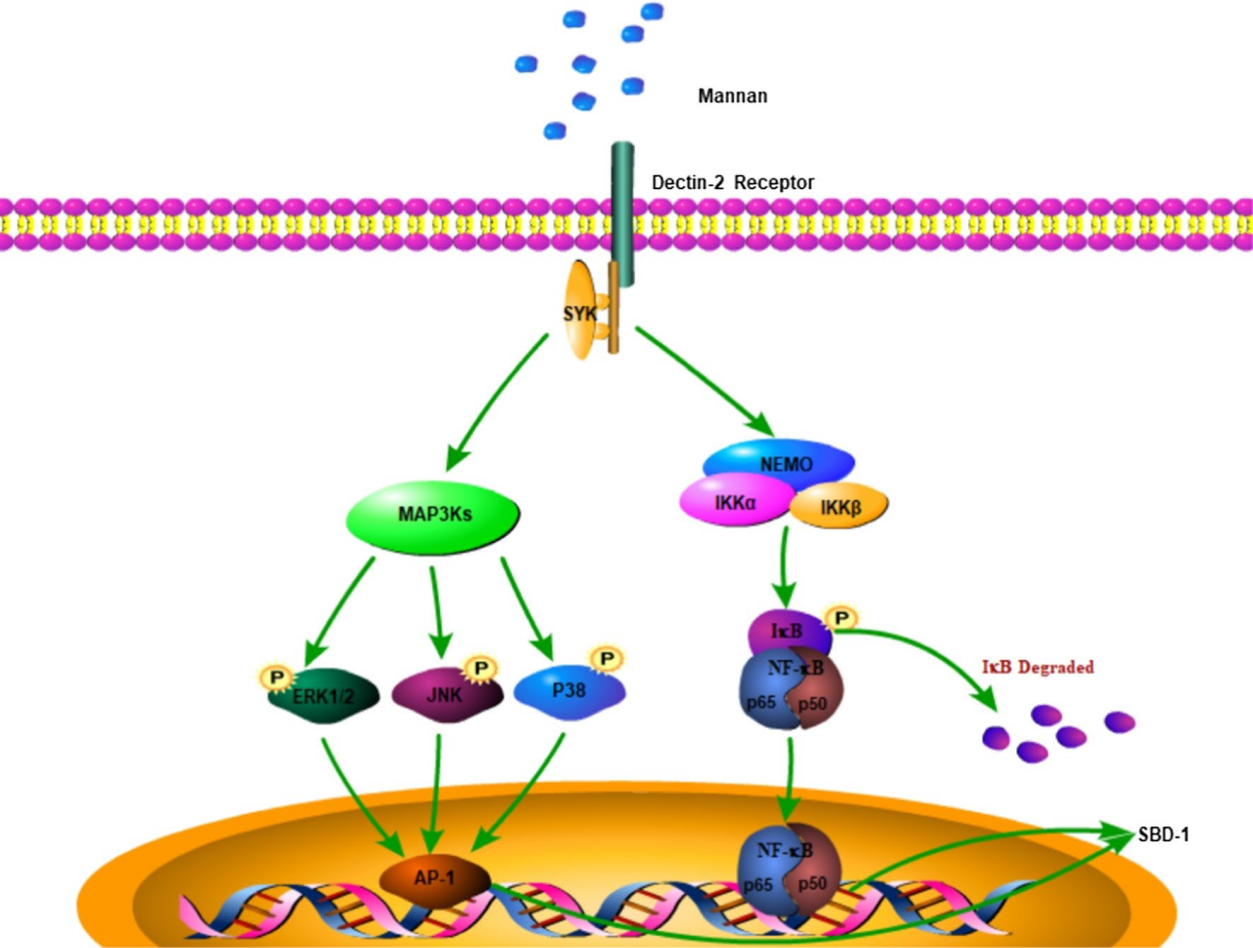
**Schematic diagram of the regulation of SBD-1 expression by mannan.** Mannan binds to Dectin-2 receptor, activates the MAPK and NF-kB signaling pathways, and subsequently stimulates SBD-1 expression in OREC.

Therefore, the mannan-induced SBD-1 expression occurs through the interaction of the Dectin-2 membrane receptor with mannan, followed by signaling to the kinase complex via the Syk adapter protein. This results in the activation of the MAPK and NF-κB signaling pathways and the transcription of the SBD-1 by a variety of proteins (Figure 8). However, the in vitro cell culture model does not represent the complex physiological environment in the body. Therefore, in future studies, we will test whether mannan regulates SBD-1 expression in vivo using animal models and whether these signaling pathways play any roles in the mannan-mediated upregulation of SBD-1 expression.

## Additional files

**Additional file 1.** **Mannan induces SBD-1 expression**. (A, B) qPCR and ELISA to assess the expression of SBD-1 after 8 h of OREC stimulation with different concentrations (10, 50, 100, 200, and 400 μg/mL) of mannan. (C) MTT assay to assess cell viability after the stimulation of OREC with different concentrations (10, 50, 100, 200, and 400 μg/mL) of mannan for 8 h. (D, E) qPCR and ELISA to detect the expression of SBD-1 after OREC were stimulated with 50 µg/mL mannan for different times (2, 4, 8, 12, 24 h). (F) MTT assay in OREC after stimulation with 50 μg/mL mannan at different times (2, 4, 8, 12, 24 h). Data are mean ± SD (*n* = 3). Different letters indicate significantly different means (*P* < 0.01).

**Additional file 2.** **Mannan induces SBD-1 expression via Dectin-2.** OREC were incubated with Dectin-2 Mouse mAb (0.1, 1, 10 μg/mL) for 30 min prior to the addition of 50 μg/mL mannan for 4 h. The SBD-1 protein expression was determined by ELISA. Data are mean ± SD (*n* = 3). Different letters indicate significantly different means (*P* < 0.01).

**Additional file 3.** **Mannan-induced upregulation of SBD-1 is Syk-dependent**. OREC were incubated with R406 (1, 5 μM) for 30 min prior to the addition of 50 μg/mL mannan for 4 h. SBD-1 protein expression was determined by ELISA. Data are mean ± SD (*n* = 3). Different letters indicate significantly different means (*P* < 0.01).

**Additional file 4.** **Effect of inhibitors on the mannan-induced SBD-1 protein expression**. OREC were cultured with mannan, with or without the SB202190 p38 inhibitor, PD98059 ERK1/2 inhibitor, SP600125 JNK inhibitor, and PDTC NF-κB inhibitor. SBD-1 expression was determined by ELISA. Data are mean ± SD (*n* = 3). Different letters indicate significantly different means (*P* < 0.01).

## Abbreviations

SBD-1: sheep β-defensin-1
OREC: ovine ruminal epithelial cells
qPCR: quantitative real-time PCR
ELISA: enzyme-linked immunosorbent assay
MAPK: mitogen-activated protein kinases
NF-κB: nuclear factor-κB
PAGE: polyacrylamide gel electrophoresis
PBS: phosphate-buffered saline
TBST: tris-buffered saline with 0.1% tween-20
FBS: fetal bovine serum
OOEC: ovine oviduct epithelial cells
