# Supplementary figures and images for: Saccharomyces cerevisiae mannan induces sheep beta-defensin-1 expression via Dectin-2-Syk-p38 pathways in ovine ruminal epithelial cells
Source: Vet Res. 2019 Feb 4;50:8. doi: 10.1186/s13567-019-0624-4 (PMC6360771; doi:10.1186/s13567-019-0624-4)

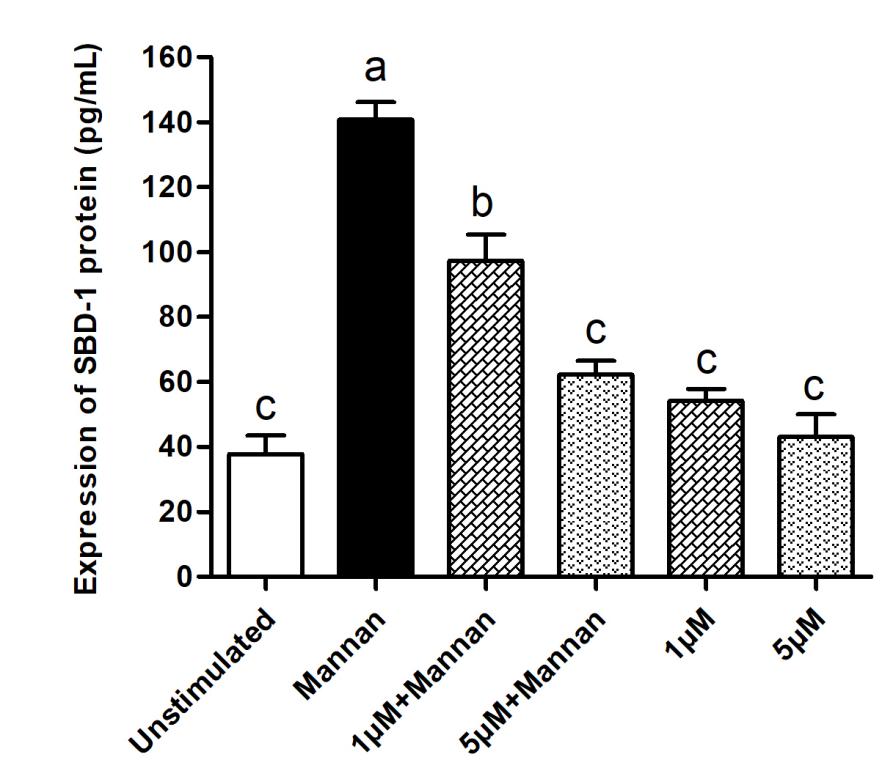

Supplement: Supplementary file 1 — Additional file 1. Mannan induces SBD-1 expression. (A, B) qPCR and ELISA to assess the expression of SBD-1 after 8 h of OREC stimulation with different concentrations (10, 50, 100, 200, and 400 μg/mL) of mannan. (C) MTT assay to assess cell viability after the stimulation of OREC with different concentrations (10, 50, 100, 200, and 400 μg/mL) of mannan for 8 h. (D, E) qPCR and ELISA to detect the expression of SBD-1 after OREC were stimulated with 50 µg/mL mannan for different times (2, 4, 8, 12, 24 h). (F) MTT assay in OREC after stimulation with 50 μg/mL mannan at different times (2, 4, 8, 12, 24 h). Data are mean ± SD (n = 3). Different letters indicate significantly different means (P < 0.01). [file 13567_2019_624_MOESM1_ESM.docx]

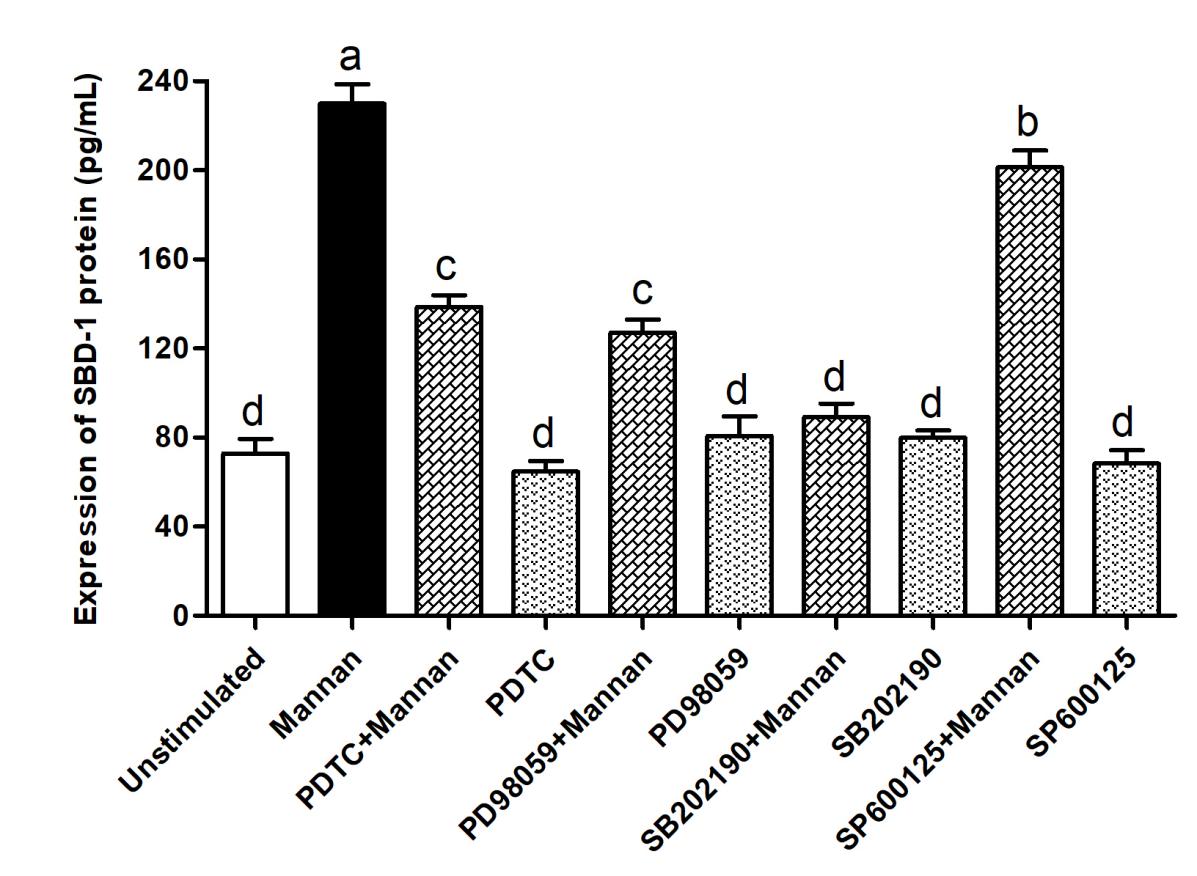

Supplement: Supplementary file 2 — Additional file 2. Mannan induces SBD-1 expression via Dectin-2. OREC were incubated with Dectin-2 Mouse mAb (0.1, 1, 10 μg/mL) for 30 min prior to the addition of 50 μg/mL mannan for 4 h. The SBD-1 protein expression was determined by ELISA. Data are mean ± SD (n = 3). Different letters indicate significantly different means (P < 0.01). [file 13567_2019_624_MOESM2_ESM.docx]

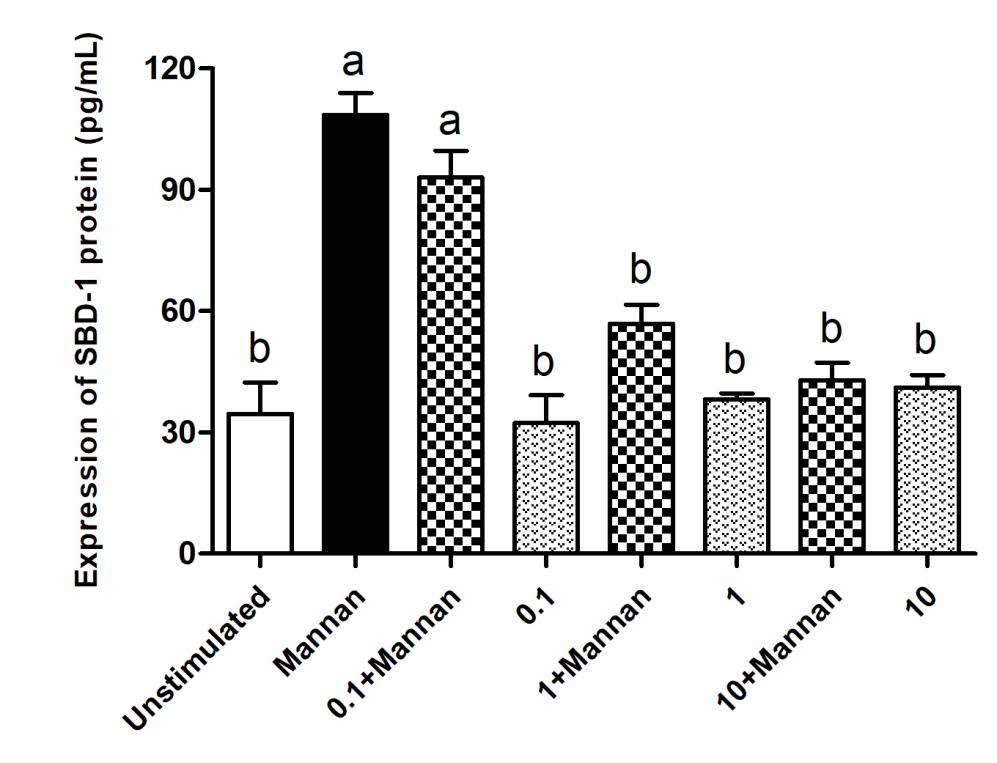

Supplement: Supplementary file 3 — Additional file 3. Mannan-induced upregulation of SBD-1 is Syk-dependent. OREC were incubated with R406 (1, 5 μM) for 30 min prior to the addition of 50 μg/mL mannan for 4 h. SBD-1 protein expression was determined by ELISA. Data are mean ± SD (n = 3). Different letters indicate significantly different means (P < 0.01). [file 13567_2019_624_MOESM3_ESM.docx]

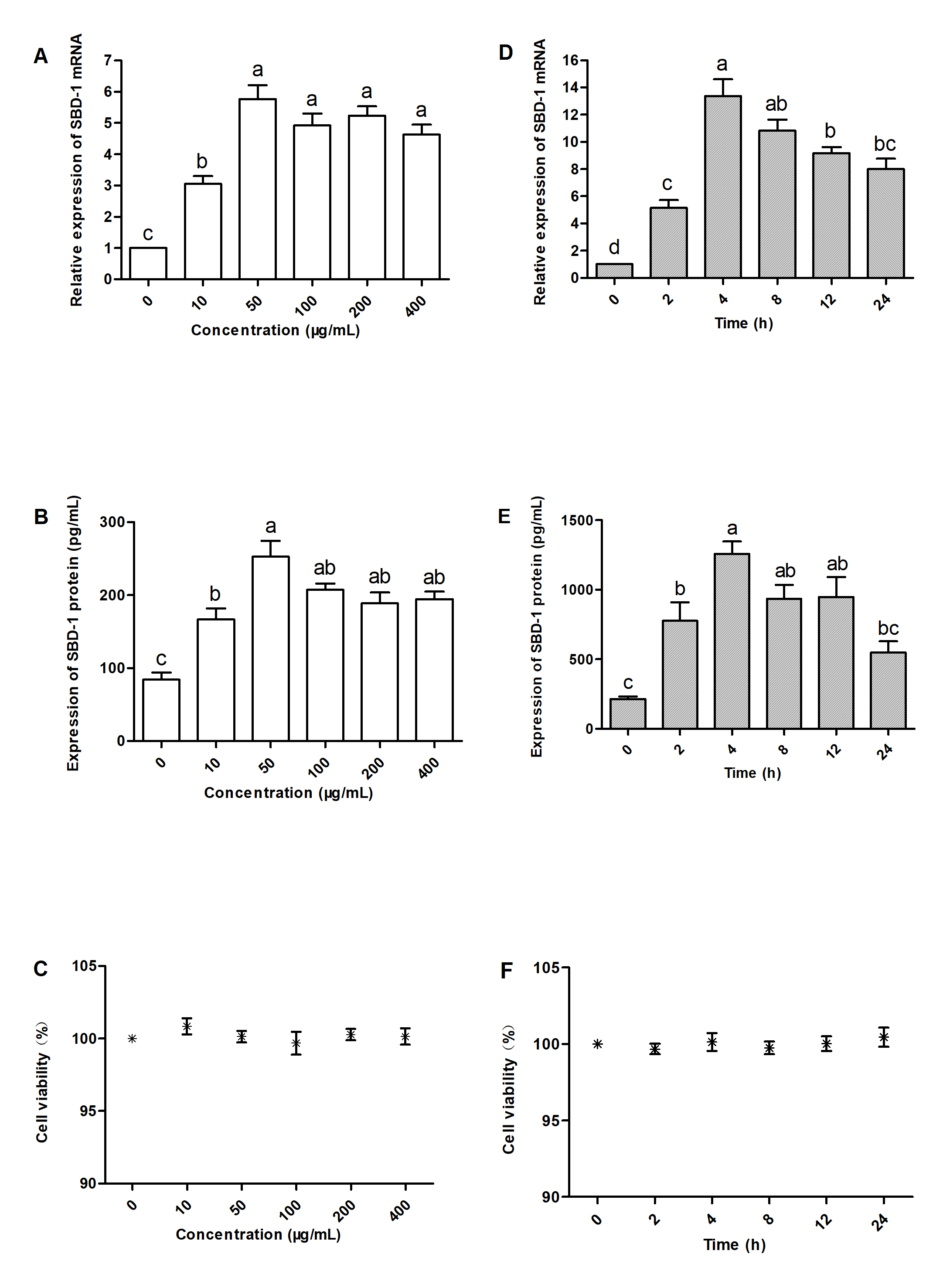

Supplement: Supplementary file 4 — Additional file 4. Effect of inhibitors on the mannan-induced SBD-1 protein expression. OREC were cultured with mannan, with or without the SB202190 p38 inhibitor, PD98059 ERK1/2 inhibitor, SP600125 JNK inhibitor, and PDTC NF-κB inhibitor. SBD-1 expression was determined by ELISA. Data are mean ± SD (n = 3). Different letters indicate significantly different means (P < 0.01). [file 13567_2019_624_MOESM4_ESM.doc]
